# Interpersonal Emotion Regulation: From Research to Group Therapy

**DOI:** 10.3389/fpsyg.2021.636919

**Published:** 2021-03-30

**Authors:** Irene Messina, Vincenzo Calvo, Chiara Masaro, Simona Ghedin, Cristina Marogna

**Affiliations:** ^1^Universitas Mercatorum, Rome, Italy; ^2^Department of Philosophy, Sociology, Pedagogy and Applied Psychology, University of Padua, Padua, Italy; ^3^Hospice “Le Rose”, Latina, Italy

**Keywords:** interpersonal emotion regulation, emotion regulation, clinical models, groups, group therapy

## Abstract

The concept of interpersonal emotion regulation (IER) refers to a variety of processes in which emotion regulation occurs as part of live social interactions and includes, among others, also those interpersonal interactions in which individuals turn to others to be helped or to help the others in managing emotions. Although IER may be a concept of interest in group therapy, specific theoretical insights in this field appear to be missed. In this article, we firstly provide a review of IER definitions, of classifications of IER strategies, and of IER clinical conceptualizations. Afterwards, we discuss the relevance of considering IER for group therapy, both in terms of non-specific group therapeutic factors and of group therapy techniques promoting adaptive emotion regulation, underlining the potentially relevant role of IER behaviors as intrinsically involved in group experience.

## Introduction

When emotions arise, individuals may use a number of processes to “influence which emotions they have, when they have them, and how they experience and express these emotions” (Gross, 1998, p. 271). Although research has mainly focused on emotion regulation considering the individual as a single isolated person, emotion regulation also involves interactive processes. Indeed, individuals often turn to others both to be helped and to reciprocally help the others in understanding and managing emotions that arise from everyday life and that involve interpersonal communication and social interaction as part of individuals' emotion regulation processes. Interpersonal emotion regulation (IER) was first mentioned by Rimé (2007), who focused on individuals' social sharing following emotional experiences. According to Rimé, IER works as an interpersonal regulatory signal that people use as emotion regulation attempts in the aftermath of an emotional experience. From Rimé's work, several interactive interpersonal processes involved in emotional management, such as social coping, social support, altruisms, and prosocial behaviors have been brought together within the IER framework; this has led to the theoretical configuration of IER as an umbrella concept including a variety of phenomena, conceptualizations, and research currents.

Interpersonal influences of emotion regulation are clearly observable in group therapy. For example, in the here-and-now of a group session, the therapist has the opportunity to observe the spontaneous manifestations of phenomena such as patients' overreliance or underuse of the group to regulate emotions, help request/provision, adoption of adaptive/maladaptive strategies to regulate internal states in relation to other group members and many others. IER may constitute a useful framework to understand and take advantage of these phenomena in group therapeutic interventions. However, theoretical developments on IER in the field of group therapy are lacking in the literature so far. With the present article, we reviewed IER definitions, classifications of IER strategies and clinical conceptualizations of IER as a potentially relevant concept for mental health, with the specific aim of providing theoretical insights on the clinical implications of IER in group therapy.

## Defining Interpersonal Emotion Regulation

A common point of all theoretical definitions is considering of IER a set of processes occurring in the context of social interactions that aim to emotion regulation. In most definitional attempts (Niven et al., 2009; Zaki and Williams, 2013; Dixon-Gordon et al., 2015; Williams et al., 2018), the motivation to modify emotions is usually emphasized because it distinguishes IER from other processes, such as emotional contagious or social coping. In fact, these processes are similar to IER in their behavioral manifestations—they occur in social interaction and involve emotional components–but they have no specific regulation goal. In this perspective, some authors have considered the presence of a regulatory goal a signal of intentionality, control, and conscious awareness of the regulatory process, affirming that implicit forms of emotional influence cannot be considered forms of IER (Niven et al., 2009; Dixon-Gordon et al., 2015). However, it should be noted that forms of emotion regulation of individuals pursuing regulatory goals in the absence of voluntary intention have been described in the case of intrapersonal regulation (Mauss et al., 2007; Koole and Rothermund, 2011). There is no reason to exclude that, in relation to unconscious emotion regulation goals, individuals may use the interaction with the others as a strategy. For example, a person may share his/her anxious feelings with a friend without being aware of the regulatory function of his/her emotional sharing.

Less consistent appears to be the definition of IER on the basis of regulation targets. If early contributions, in line with traditional models of regulation, considered the self the unique target of emotion regulation (Rimé, 2007; Marroquín, 2011), more recent contributions have extended the concept also to extrinsic aspects of emotion regulation, considering the other person the target (Niven et al., 2009; Zaki and Williams, 2013). An example of extrinsic IER may be represented by providing comfort in order to regulate another person's sadness. This extension of IER to extrinsic regulation has weakened the theoretical boundaries of the concept, including empathic, supportive, and prosocial behaviors within the concept of IER in all cases in which these processes have regulatory goals (Zaki, 2019).

Another element considered in IER definition is the difference with traditional intrapersonal regulation. Intrapersonal and interpersonal aspects of emotion regulation can be viewed as part of the continuum of self-involvement in regulatory processes. On one extremity, we find self-regulation processes (intrapersonal regulation) and, on the other extremity, we find the absence of self-involvement in regulatory processes (regulation by others or regulation of others), with co-regulation in the halfway position (Campo et al., 2017). For this reason, in some cases, it is not possible to establish a clear boundary between intrapersonal and interpersonal regulation processes. For example, a person may intrapersonally reappraise a situation using recalling a suggestion provided by a friend in past situations, or he/she may suppress emotional reactions on the basis of parental education. Several authors, therefore, adopted an operational definition of IER as regulatory processes that happen in the context of live social interactions (Zaki and Williams, 2013; Williams et al., 2018). Maintaining an operational focus, though the importance of co-regulatory processes is widely recognized in IER literature, the complex dynamic of reciprocal influences is supposed to be better understood using emotional system theories and methodologies (Butler et al., 2014).

## Interpersonal Emotion Regulation Strategies

People may use different strategies for emotion regulation in social interactions. In clinical psychology, early theoretical efforts aimed to the classification of IER strategies extended traditional models of intrapersonal emotion regulation to interpersonal regulatory phenomena. With reference to the traditional Gross's process model, Marroquín (2011) has listed a series of interpersonal influences that may occur when considering attention deployment and cognitive change steps of the emotion regulation process. According to Marroquín, when considering the step of attention deployment, the others may intervene in one person's emotion regulation process distracting the person from a situation, for example, by reorienting the person to neutral/positive stimuli, by providing neutral/positive stimuli, or by helping the person to focus on concrete or non-self-relevant stimuli. In the step of cognitive change, interpersonal influence may involve the generation and the selection of alternative interpretations, the highlight or supply of schema-inconsistent information, the explicit correction of cognitions, and the addition of flexible processing resources. Christensen and Haynos (2020), also starting from Gross's model, have conceptualized IER as strategies involving situation selection or situation modification (for example, IER strategies helping individuals to avoid exposure to situations that elicit an emotional response or helping individuals to change that situation), as well as response modulation (for example, expressive suppression may be used to deal with perceived social concerns about the appropriateness of one's expressed emotions).

Zaki and Williams (2013) introduced the distinction between response-dependent IER that requires particular qualities of another person's response (for example, after emotional sharing the person may receive support depending on the response of the other), and response-independent IER, which does not require a particular response from the other person (for example, labeling the emotion as effective regardless of the others' response).

An empirically based classification of IER strategies was provided when creating the *Interpersonal Emotion Regulation Questionnaire* (IERQ; Hofmann et al., 2016), which evaluates the ways a people use others to regulate his/her own emotions (intrinsic IER). Interestingly, a qualitative data analysis was used to generate the items and to create an empirically based IER model. The results was a 20-item questionnaire evaluating the following subscales: (a) Enhancing positive affect, which describes the tendency to seek out the others in order to increase feelings of happiness and joy (item example “Because happiness is contagious, I seek out other people when I'm happy”); (b) Perspective taking, which involves the use of others in order to be reminded not to worry and that others have it worse (item example: “Having people remind me that others are worse off helps me when I'm upset”); (c) Soothing, which consists in seeking out the others for comfort and sympathy (item example: “I look to others for comfort when I feel upset;” (d) Social modeling, concerning looking to others to see how they might cope with a given situation (item example: “If I'm upset, I like knowing what other people would do if they were in my situation”).

In the field of developmental psychology, a list of adaptive and maladaptive extrinsic IER strategies has been provided by Pacella and López-Pérez (2018) as part of the implementation of an online serious game that evaluates how children engage in modifying the emotions of others. In this list, they include positive affective engagement, cognitive engagement, distraction and humor as adaptive strategies, and suppression, co-rumination, avoidance, diminishing comparisons, and negative behavioral engagement as maladaptive strategies.

## Clinical Models of Interpersonal Emotion Regulation

Early theoretical contributions in the field of IER have considered its implication for emotional disorders conceptualization and treatment (Marroquín, 2011; Hofmann, 2014; Christensen and Haynos, 2020), assuming the key role of IER as a mediator factor in the widely described negative association between depression and social support (Marroquín, 2011). According to this view, depression is negatively influenced by the lack of opportunities to interpersonally regulate emotions in socially supporting context, and this problem plausibly concerns any psychopathology that is influenced by social isolation.

Subsequent contributions have observed both the positive and the negative consequences of IER for psychopathology. Hofmann (2014) theorized that IER strategies can be a protective factor for anxiety and mood disorders at the extent to which they weaken the effects of emotional distress but, on the other hand, they can also perpetuate psychopathological symptoms, such as in the case of one's exaggerated dependency on others to regulate his/her own emotions. The issue of dependency/autonomy imbalance in regulation behaviors calls into consideration clinically relevant contributions of developmental psychology. Recent findings have revealed significant age effects in extrinsic IER, showing that older children and adolescent use more adaptive and more sophisticated/various extrinsic regulation strategies compared to younger children (López-Pérez et al., 2016; Pacella and López-Pérez, 2018; Gummerum and Lopez-Perez, 2020; López-Pérez and Pacella, 2021). In line with developmental views of psychopathology, maladaptive forms of IER can be attributed to deficiencies in individual development related to interpersonal components of emotion regulation in early relationships (Mikulincer et al., 2003; Shaver and Mikulincer, 2007; Messina et al., 2016a).

Dixon-Gordon et al. (2015) have listed failures that could occur in different steps of IER processes, causing peoples' dysregulation. According to the authors, in the case of intrinsic IER, failures in emotion regulation may be related to the overreliance on others to regulate emotions, underuse of social environment to regulate emotions, selection of inappropriate or unhelpful others, overreliance on particular individuals, unavailability of others to regulate emotions, or selection of inappropriate settings. About extrinsic IER, failures in regulating others' emotions may be related to excessive attempts or failures to regulate others' emotions or selection of inappropriate settings for emotion regulation.

A focus on IER clinical features has been provided also in the construction of the questionnaire *Difficulties in Interpersonal Emotion Regulation* (DIRE; Dixon-Gordon et al., 2018), which evaluates the relevance of IER strategies in psychopathology. The questionnaire presents a series of scenarios and asks the individuals to indicate the likelihood according to which they would respond to each scenario referring to the listed ways, which include intrinsic IER forms together with some intrapersonal emotion regulation strategies such as distraction and avoidance. The items describing each strategy were generated on the basis of previous theories and research and involved the following strategies: talking about one's emotions, seeking reassurance, seeking problem-solving support, and venting. The factorial analysis revealed two factors: the first factor included reassurance-seeking items (item example “keep asking for reassurance”) and the second factor included venting items (item example “raising voice or complaining”). Both factors were associated to negative mental health outcomes.

With regard to empirical investigation of IER in the clinical context, early quantitative studies have found IER peculiarities in psychopathological sample. For example, in cases of anxiety and depression (Altan-Atalay and Saritas-Atalar, 2019), of borderline personality disorder (Dixon-Gordon et al., 2016; López-Pérez et al., 2017), and of substance addiction (Dingle et al., 2018), individuals have appeared to have significantly different IER behaviors.

Diary-based studies of romantic partner relational dynamics have provided interesting insight regarding positive and negative consequences of IER. For example, touch (Debrot et al., 2013) and humor (Horn et al., 2019) have emerged to be effective forms of IER. Positive and negative consequences of IER in couples have been investigated also considering their association with emotional disorder symptoms. Horn and Maercker (2016) have considered the effects of co-reappraisal (cognitively changing a situation's meaning during a conversation with the partner) and co-brooding (passive repetitive focus on negative content, which is unwanted, rigid and perceived as unpleasant during a conversation with the partner) on three different symptoms of maladjustment: preoccupation, failure to adapt, and depression. Co-brooding was a significant predictor of all maladjustment symptoms, whereas co-reappraisal was predictive of less depressive symptoms and lower adjustment to the disorder symptoms in the female sample. Thus, IER seems to have an important role in mitigating or intensifying the severity of emotional disorders.

## Which Implications for Group Therapy?

Interventions directly targeting emotion-regulation skills have been largely encouraged by clinical psychology literature (Berking et al., 2008; Messina et al., 2016b, 2020; Frederickson et al., 2018; Grecucci et al., 2020). Referring to the social dimension of emotion regulation, interventions aiming to improve emotion-regulation skills appear to be potentially relevant. Therefore, group therapy may be a promising context in the work on the interpersonal features of emotion regulation (and dysregulation).

On the basis of the extant state of art on IER conceptualization and empirical research, early applications of IER conceptualizations can be contextualized in group therapy. First, regardless of the explicit therapeutic purpose of the group, the experience of being part of a group could have therapeutic effects, as well as it is observed in self-help or psychological support groups (Marogna and Caccamo, 2014). Several aspects of group experiences may have therapeutic implications. First, the group is a natural source of social support. Second, altruism has also been recognized as a therapeutic group factor to the extent that in groups, patients find real opportunities to be helpful to others (extrinsic regulation). Thus, the group experience implies potential sources of corrective relational experiences when facing negative experiences in the outside world (Caccamo et al., 2017, 2018).

Second, the therapist may promote the use of constructive IER strategies during the course of the group therapy. Among numerous possibilities, examples of therapists' interventions aiming to promote IER within group therapy experience are the following:

- he/she can invite the group members to share emotions (social sharing);
- he/she can invite the group members to provide alternative interpretations of individual points of view (cognitive change/perspective taking);
- he/she can invite the group members to share their own experiences in coping with the emotional experiences reported by another member (social modeling).

Third, the therapist may discourage the use of dysfunctional IER strategies (such as venting), focusing the group attention on the relational consequences and on the emotional outcomes, promoting the group discussion (and awareness) of more appropriate alternative strategies.

Fourth, the group is an optimal context to observe the spontaneous manifestations of IER phenomena in the interactions among group members. The therapist may stimulate individual and group awareness concerning the use of IER strategies. In this regard, he/she can:

- point out regulatory attempts related to behavioral manifestations;
- promote the reflection on positive/negative consequences of IER behaviors in terms of relational quality and emotional outcomes;
- point out social phenomena such as overreliance/underreliance on others to regulate emotions (how much the person use the group, the therapist, or a specific group member to regulate his/her emotions?)
- point out phenomena such as selection of inappropriate or unhelpful others (for example, reflecting on different outcomes of IER that the person may have in the group compared with dysfunctional everyday life relations);
- point out the selection of inappropriate moments (for example, asking for help during another member's important moment of social sharing).

Finally, all the described strategies may be effectively adopted only when a suitable context for effective emotion regulation is available. In this perspective, the therapist may have a key role in establishing a positive atmosphere of acceptance, respect, and non-judgment regarding emotional expression and regulation attempts from group members. Indeed, emotion regulation is not only a matter of strategy: it also implies a sense of curiosity about emotions, a perspective that does not consider emotions and thoughts as threats, but rather as mental phenomena which are precious sources of information on one's current mental state.

## Outstanding Issues and Future Developments

The definitions of IER reviewed in the present article have provided a useful basis for the conceptualization of IER phenomena in group therapy. Several aspects of available IER strategy classifications and clinical models may help group therapists in recognizing aspects of group functioning which may be potentially relevant in therapy sessions. However, classification of IER strategies specific for group therapy context should be provided in future works. With regard to empirical investigations on IER, group therapy research is still missing. Both quantitative and qualitative studies have offered interesting insights on IER phenomena, but only specific forms of regulation and specific pathological samples have been investigated. Extant research on IER actually appears to be scattered and limited in providing concrete clinical implications, and we are far from having a comprehensive empirically founded perspective of IER to be used for clinical practice. In this context, a positive starting point is the availability of standardized and non-standardized scales to evaluate IER in adults, as well as of new promising methodologies for the assessment of IER in the developmental age which may both offer a strong basis for future research on IER. In particular, the employment of IER scales could be useful in future research investigating the potential mediating role of IER in the recognized link between attachment style, dyadic adjustment, and individual well-being (Calvo and Bianco, 2015; Calvo et al., 2015, 2020; Ghedin et al., 2017).

## Author Contributions

IM contributed to conception and wrote the first draft of the manuscript. SG and CMar contributed to literature review and article collection. VC and CMas wrote the final version of the manuscript. All authors contributed to manuscript revision, read, and approved the submitted version.

## Conflict of Interest

The authors declare that the research was conducted in the absence of any commercial or financial relationships that could be construed as a potential conflict of interest.
